# Trigger Twins: 2 Cases of Ipsilateral Twin Trigger Digit and a Review of Published Literature

**DOI:** 10.1155/2019/8697360

**Published:** 2019-06-17

**Authors:** David Brinkman, Gerard Sheridan, Michael O'Sullivan

**Affiliations:** Department of Orthopaedics, Galway University Hospital, Newcastle Road, Galway, Ireland

## Abstract

**Background:**

The aetiology of trigger digits has been debated since Notta first described them in 1850. Aetiology has been segregated for adult and paediatric presentations. While an adult trigger digit is most likely an acquired inflammatory process, the same is not true for cases in children: no inflammatory reaction is seen on microscopic evaluation. We wish to add strength to a genotypical aetiology for paediatric cases.

**Methods:**

We present two cases of monozygotic twins with trigger digits managed in our institution. A comprehensive review of literature was conducted for cases of trigger digit in monozygotic twins to support our theory.

**Results:**

Our two sets of twins enjoyed a full recovery from surgery at one-month follow-up. A total of seven other cases of monozygotic twins were found in literature.

**Conclusion:**

Our cases add to the growing body of evidence supporting a genotypical aetiology for paediatric trigger digit.

## 1. Introduction

In 1850, Notta first described a series of patients with a nodule on a tendon that was interfering with its gliding [1]. His name has been attributed to the nodule often palpated on the flexor aspect of the digit affected by trigger thumb or trigger finger, a bundle of mature collagen and fibroblasts along the flexor tendon [2]. Palpation of same and the presence of “triggering” movement of the distal joint lead to the clinical diagnosis. Since its description, the aetiology has been a topic of much debate.

While trigger digit in adult populations is known to be an inflammatory process and there have been reports of inflammatory trigger digit cases in older children with extensive use of their hands, the pathological process of younger paediatric cases of trigger digit is unknown. In children with no history of trauma or heavy use of hands, we propose a genetic aetiology. To support this, we present the first set of twins with ipsilateral trigger thumbs in literature and the first set of twins with a 3^rd^ sibling to present to the orthopaedic service with the same condition. We include a review of the literature to further support the theory.

## 2. Materials and Methods

### 2.1. Case 1

Our first set of monozygotic male twins presented at 6 years and 6 months old. Twin 1 had bilateral trigger thumb; twin 2 had a trigger thumb and contralateral trigger finger. The twins were treated with A1 pulley release, including flexor digitorum superficialis slip release of the digit. One month later, their sister of 4 years, 9 months old presented with a single trigger thumb and is currently being managed conservatively. None of the patients had a history of trauma and had normal motor and sensory function in affected digits.

### 2.2. Case 2

**Figure 1:**
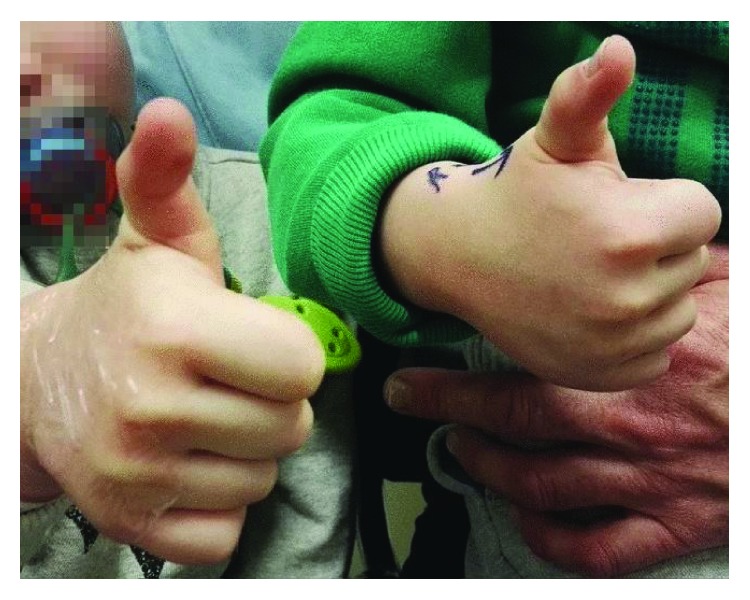
Ipsilateral trigger thumbs.

**Figure 2:**
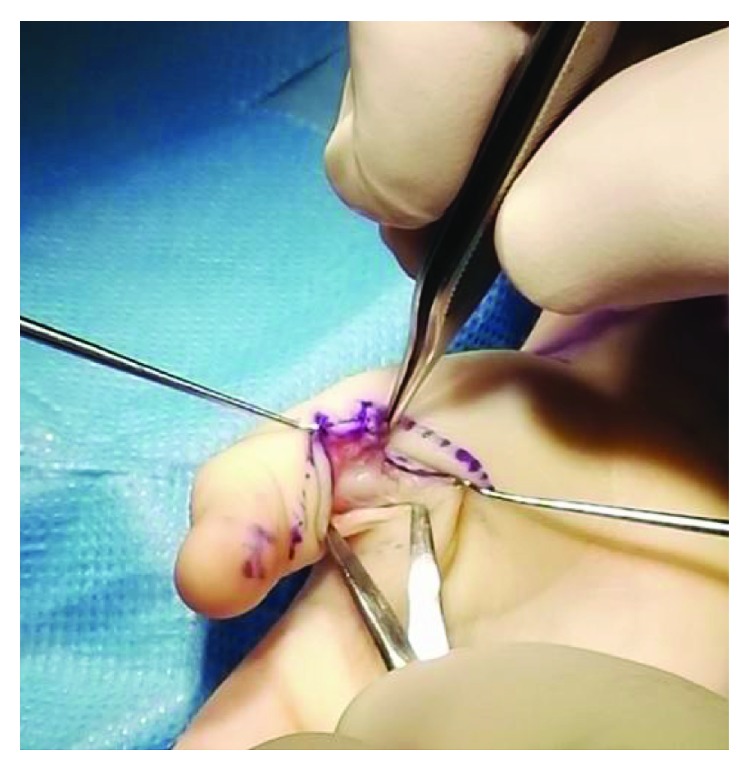
Node of Notta visible during A1 pulley release.

Monozygotic male twins presented at 3 years, 4 months old with right-sided trigger thumb (Figure 1). Both twins were treated with bilateral A1 pulley release (Figure 2). Again, neither of the patients had a history of trauma and had normal motor and sensory function in affected digits.

### 2.3. Literature Review

A comprehensive review of literature was conducted using PubMed using the terms “trigger (thumb OR digit)” and “twin”. Article abstracts were screened as follows: articles that reported cases of trigger digits in monozygotic twins, published in English, and were included for review. Notable exclusion criteria included articles reporting solely siblings and other family members. Subject-specific review articles' and suitable case report articles' references were also screened for appropriate articles to include.

## 3. Results

### 3.1. Patient Outcomes

Both sets of twins enjoyed return to full normal function at 1-month follow-up. There was no requirement for physiotherapy for this intermediate procedure in a paediatric population. The wounds healed fully and did not impair function. They were discharged to the community physician.

### 3.2. Review of Literature

Our search of literature found 87 unique articles. 85 articles were found to be unsuitable for inclusion. A further set of 5 articles was found to be suitable for inclusion on review of references of case reports and relevant review articles. In total, 7 cases of paediatric trigger digits have been reported in monozygotic twins in the literature. These included 5 cases of trigger thumbs alone: two bilateral sets [3, 4], two contralateral sets [5, 6], and one case of twins, one with a unilateral trigger thumb, the other with bilateral trigger thumb [7]. One case of twins with simultaneous trigger thumbs and digits was reported [8]. Another case describes twins with first interphalangeal joint triggering only, one twin having unilateral disease, the other bilateral [9].

The average age at presentation of all cases was 3 years and 1 month with a standard deviation of 1 year and 8 months. Sex was reported in 7 of the cases, with a 5 : 2 male to female ratio.

## 4. Discussion

Trigger thumb and trigger finger present similarly; however, due to their differing anatomical constructs, they require different management. While trigger thumb is due to A1 pulley constriction, trigger finger can be constriction of A1, A2, or A3 pulleys, flexor digitorum profundus (FDP) or FDS thickening, an abnormal relationship between the two or tendons or proximal FDS decussation. Trigger thumb is treated adequately with a simple A1 pulley release [10], whereas trigger finger requires an additional resection of a slip of FDS for release [11].

The aetiology of trigger finger and thumb is unknown as of yet. Unlike adult trigger finger which is inflammatory in origin, paediatric trigger thumb and finger have no such features [2]. There are, however, multiple theories: an abnormal arrangement of the sesamoids causing bunching up of the spiral fibres of collagen [12], subtle trauma [13], or a constant flexed position in utero causing collagen degeneration and synovial proliferation [14]. The above collection of case reports of monozygotic twins presented suggests a familial element to these aetiologies. Interestingly, all suitable case reports found of monozygotic twins were in the paediatric population, our case of 6½ year olds being the oldest. There is a male preponderance in the reported cases.

Further study of aetiology is made difficult by its low incidence. Epidemiological studies report an incidence of 3.3 trigger thumbs in 1000 at 1 year old in Japan [15] and no reports of sibling incidences or epidemiological studies of trigger thumb, presumably due to low incidence.

## 5. Conclusion

A multifactorial aetiology to this condition is supported by literature. While there is no clear pattern of digits involved, a hereditary and genetic element is likely given the number of monozygotic twins, siblings, and generations [16, 17] reported in the literature. Further research into the proposed causes of toddler trigger digits is required to prove this theory.
